# Supplementary material for: Non-canonical signalling mediates changes in fungal cell wall PAMPs that drive immune evasion
Source: Nat Commun. 2019 Nov 22;10:5315. doi: 10.1038/s41467-019-13298-9 (PMC6876565; doi:10.1038/s41467-019-13298-9)
Supplement: Supplementary file 1 — Supplementary information [file 41467_2019_13298_MOESM1_ESM.pdf]

Supplementary Information

Pradhan *et al.*

CONTENTS:

Supplementary Table 1. *C. albicans* strains

Supplementary Figure 1. Impact of iron on β-glucan exposure in *C. albicans*.

Supplementary Figure 2. Effects of db-cAMP on β-glucan masking mutants.

Supplementary Figure 3. Iron limitation-induced masking affects chitin content.

Supplementary Figure 4. Gating strategy for cytometry experiments.

Supplementary References

Supplementary Table 1. *C. albicans* strains

Strain	Origin/ pseudonym	Parent	Genotype	Source
SC5314	blood		clinical isolate: clade 1	Gillum *et al.* (1984) [1]
IHEM16614	oropharynx		clinical isolate: clade 2	Donna MacCallum
J990102	vagina		clinical isolate: clade 3	MacCallum *et al.* (2009) [2]
AM2005/0377	oral cavity		clinical isolate: clade 4	Donna MacCallum
CAF2-1	CAF2-1	SC5314	*ura3Δ::imm434/URA3*	Fonzi & Irwin (1993) [3]
CAI4	CAI4	CAF2-1	*ura3Δ::imm434/Δura3Δ::imm434*	Fonzi & Irwin (1993) [3]
Ca372	CAI4+CIp10	CAI4	CAI4, *RPS1-CIp10 (URA3)*	Murad *et al.* (2000) [4]
RM1000	RM1000	CAI4	*ura3Δ::imm434/Δura3Δ::imm434, his1Δ::hisG/his1Δ::hisG*	Negredo *et al.* (1997) [5]
Ca674	RM1000+CIp20	RM1000	RIM1000*, RPS1-CIp20 (URA3,HIS1)*	Smith *et al.* (2004) [6]
BWP17	BWP17	RM1000	*ura3Δ::imm434/Δura3Δ::imm434, his1Δ::hisG/his1Δ::hisG, arg4Δ::hisG/arg4Δ::hisG*	Wilson *et al.* (1999) [7]
Ca1206	BWP17+CIp30	BWP17	BWP17*, RPS1-CIp30 (URA3,HIS1,ARG4)*	Dennison *et al.* (2005) [8]
DAY185	DAY185	BWP17	BWP17, *his1::hisG::HIS1*	Davis *et al.* (2000) [9]
SN148	SN148	CAF2-1	*arg4Δ/arg4Δ, leu2Δ/leu2Δ, his1Δ/his1Δ, ura3Δ::imm434/ura3Δ::imm434, iro1Δ::imm434/iro1Δ::imm434*	Noble & Johnson (2005) [10]
SN152	SN152	CAF2-1	*arg4Δ/arg4Δ, leu2Δ/leu2Δ, his1Δ/his1Δ, URA3/ura3Δ::imm434, IRO1/iro1Δ::imm434*	Noble & Johnson (2005) [10]
LR2	*gpr1Δ*	CAI4	CAI4, *gpr1 ::hisG/gpr1 ::hisG-URA3-hisG*	Maidan *et al.* (2005) [11]
NM6	*gpa2Δ*	CAI4	CAI4, *gpa2 ::hisG/gpa2 ::hisG-URA3-hisG*	Maidan *et al.* (2005) [11]
NM23	*gpr1Δ gpa2Δ*	CAI4	CAI4, *gpa2Δ::hisG/gpa2Δ::hisG, gpr1Δ::hisG/gpr1Δ::hisG-URA3-hisG*	Maidan *et al.* (2005)[11]
CR323	*cyr1Δ* (*cdc35Δ*)	CAI4	CAI4, *cdc35Δ::hisG/cdc35Δ::hisG, pVEC-URA3*	Rocha *et al.* (2001) [12]
ftr1	*ftr1Δ*	CAI4	CAI4, *ftr1Δ::hisG/ftr1Δ::hisG*	Ramanan & Wang (2000) [13]
tpk1Δ	*tpk1Δ*	SN152	SN152, *tpk1::HIS1/ tpk1::ARG4*	Cao *et al.* (2017) [14]
tpk2Δ	*tpk2Δ*	SN152	SN152, *tpk2::HIS1/ tpk2::ARG4*	Cao *et al.* (2017) [14]
tpk1Δ tpk2Δ	*tpk1Δ tpk2Δ*	SN152	SN152, *tpk1::LEU2/ tpk1::FRT, tpk2::HIS1/ tpk2::ARG4*	Cao *et al.* (2017) [14]
GOA31	*goa1Δ*	SN148	SN148, *goa1Δ::URA3/goa1Δ::ARG4*	Bambach *et al.* (2009) [15]
CA-IF003	*sod1Δ*	SN152	SN152*, sod1Δ::cmLEU2/sod1Δ::cdHIS1*	Frohner *et al.* (2009) [16]
CA-IF007	*sod2Δ*	SN152	SN152*, sod2Δ::cmLEU2/sod2Δ::cdHIS1*	Frohner *et al.* (2009) [16]
CA-IF011	*sod3Δ*	SN152	SN152*, sod3Δ::cmLEU2/sod3Δ::cdHIS1*	Frohner *et al.* (2009) [16]
CA-IF070	*sod4/5/6Δ*	SN152	SN152, *sod5Δ::cmLEU1/sod5Δ::cd HIS1, sod4Δ::FRT/sod4Δ::FRT, sod6Δ::FRT/sod6Δ::FRT*	Frohner *et al.* (2009) [16]
sef1	*sef1Δ*	SN152	SN152, *sef1Δ::cmLEU2/sef1Δ::cdHIS1*	Noble *et al.* (2010) [17]
sfu1	*sfu1Δ*	SN152	SN152, *sfu1Δ::cmLEU2/sfu1Δ::cdHIS1*	Noble *et al.* (2010) [17]

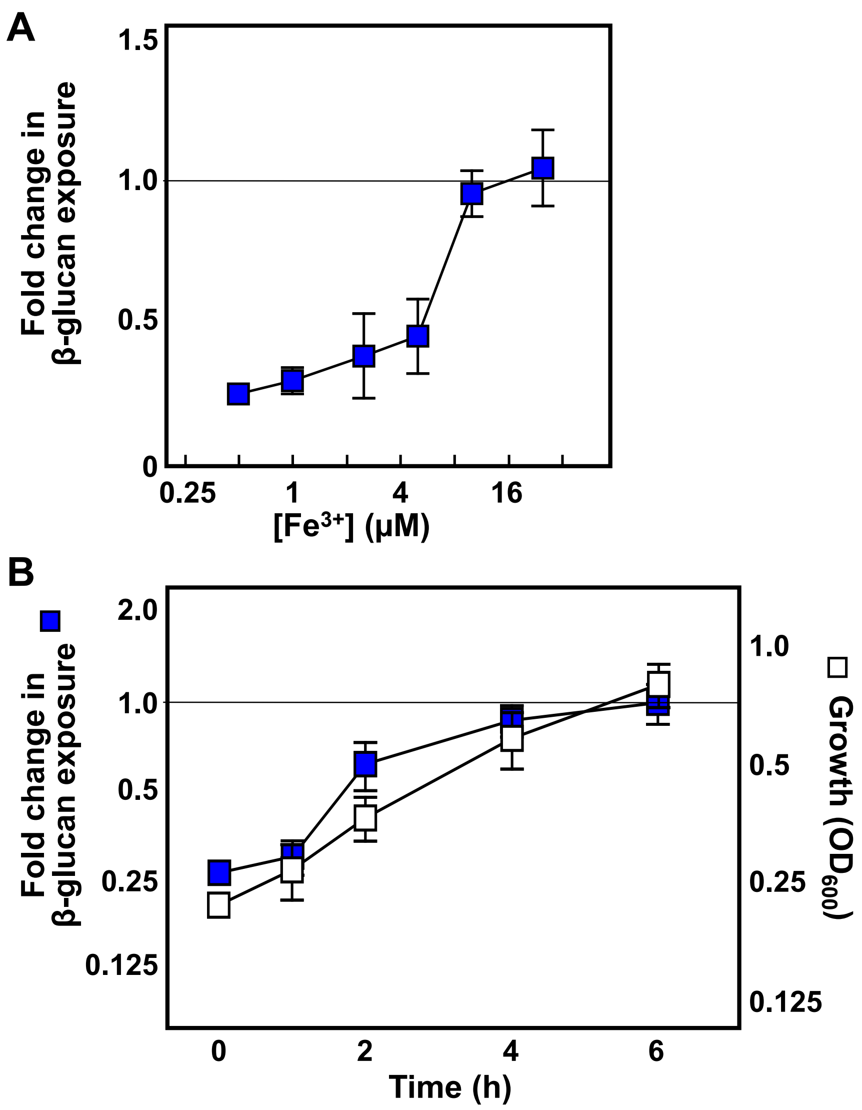

Supplemental Figure 1: Impact of iron on β-glucan exposure in *C. albicans*. (A) Dose-dependent β-glucan masking. Wild type *C. albicans* cells (SC5314: Supplementary Table 1) were exposed to different concentrations of $FeCl_3$ 5 h, fixed and their levels of β-glucan exposure quantified by Fc-dectin-1 staining and cytometry, as described in Fig. 6. **(B)** *C. albicans* SC5314 cells were grown under iron limiting conditions, and then transferred to iron replete conditions (Methods). At various times thereafter, their growth (OD_{600}; white squares) and levels of β-glucan exposure were quantified as described above (blue squares). Means and standard deviations from three independent replicate experiments are shown.

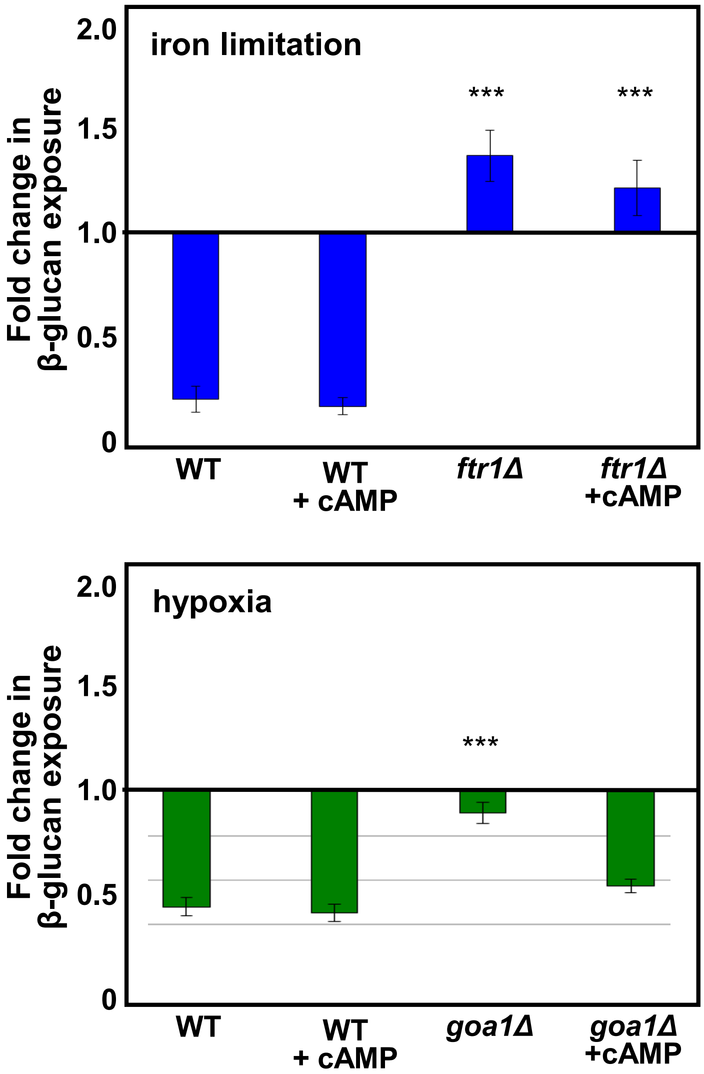

Supplemental Figure 2: Exogenous db-cAMP suppresses the hypoxia-induced masking defect of *goa1Δ* cells, but not the iron limitation-induced masking defect of *ftr1Δ* cells. The same batch of db-cAMP was used in these parallel experiments. These new hypoxia data recapitulated our earlier observation that db-cAMP suppresses the defect in hypoxia-induced β-glucan masking defect of *C. albicans goa1Δ* cells [28]. See Supplemental Table 1 for strain details. Means and standard deviations from three independent replicate experiments are shown, and the data were analysed using ANOVA with Tukey's multiple comparison test: *, $p < 0.05$; **, $p < 0.01$; ***, $p < 0.001$. Source data are provided as a Source Data file.

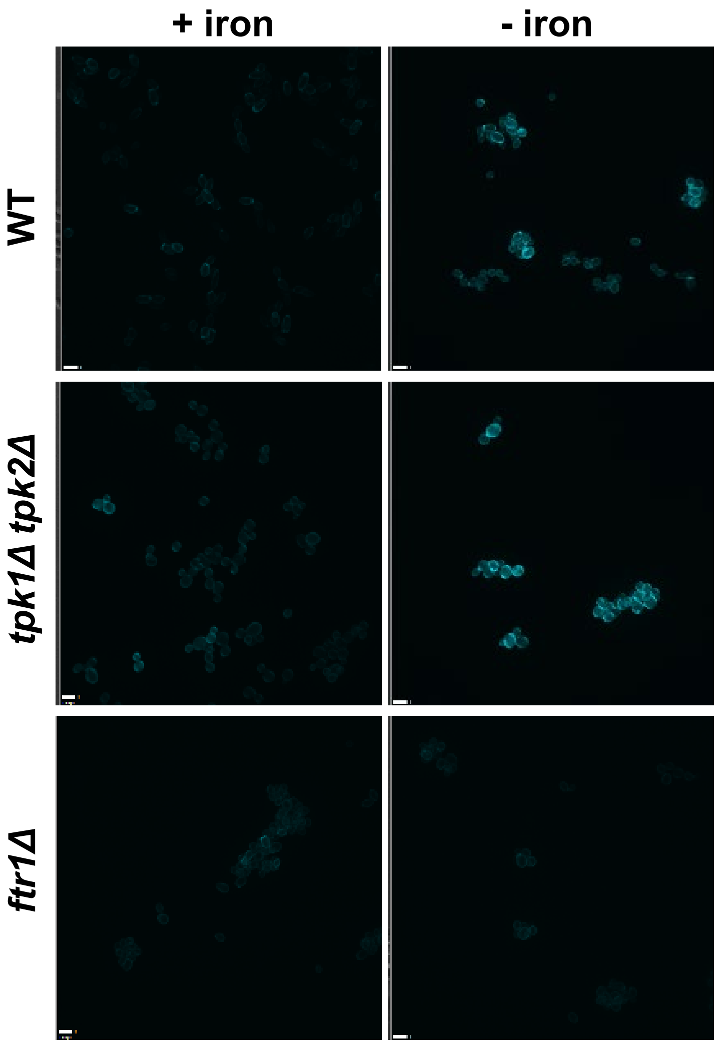

Supplemental Figure 3: Fluorescence microscopy of Calcofluor White-stained *C. albicans* cells following iron limitation-induced masking. *C. albicans* cells were cultured under iron replete or limiting conditions (Methods), fixed, and their chitin stained with Calcofluor White (10 μg mL^{-1}). These stained cells were subjected to fluorescence microscopy (scale bars 5 μm): WT, wild type, SC5314; *tpk1Δ tpk2Δ*; *ftr1Δ* (Supplementary Table 1).

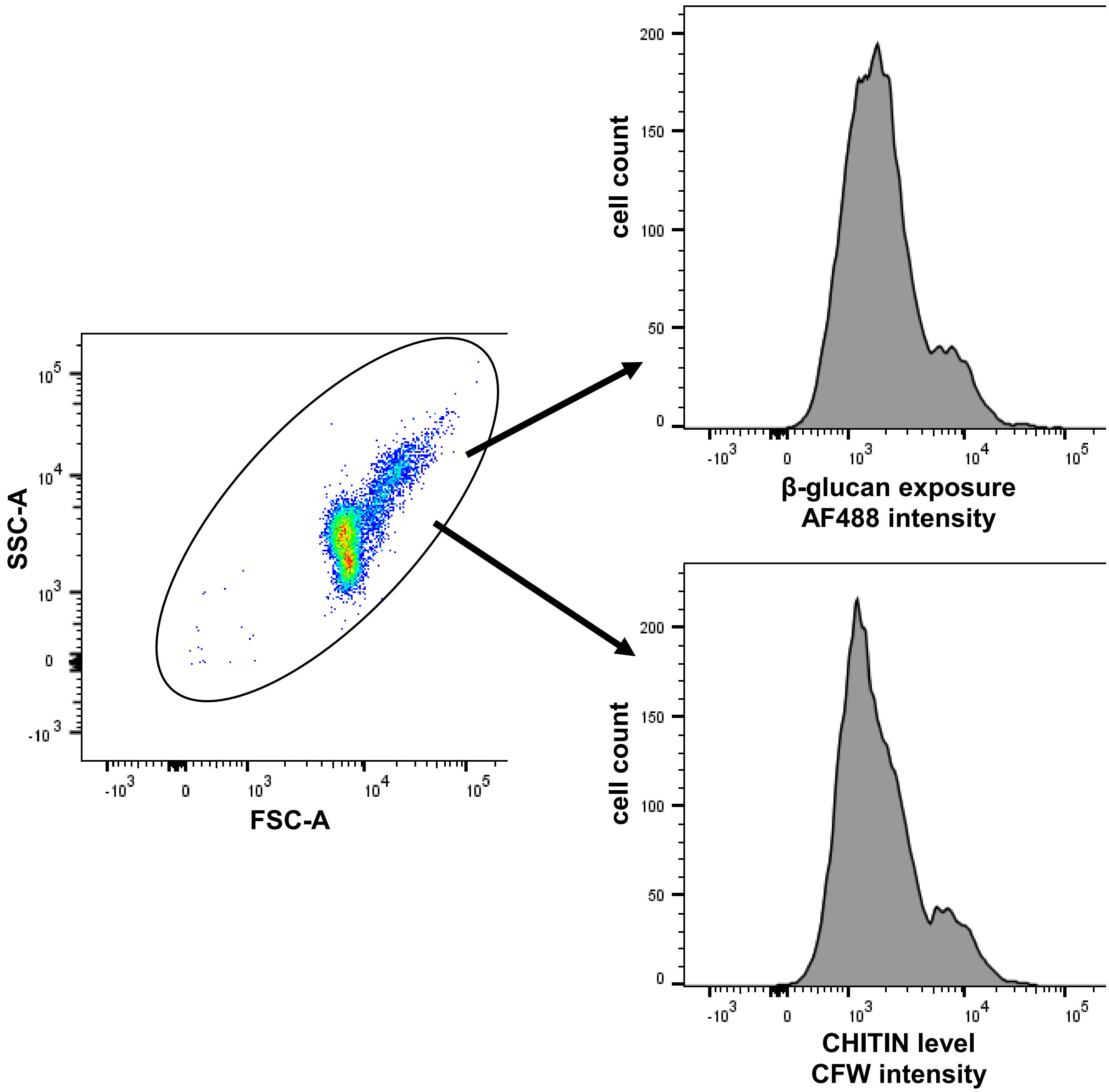

Supplemental Figure 4: Gating strategy for cytometry experiments. *C. albicans* populations were simply gated on the basis of the scatter plot (FSC/SSC), and the gated population analyzed for AF488 or CFW fluorescence intensity. The axes shown in this figure are the same as for all figures in the paper, and these axes remained unchanged throughout.

Supplementary References

1. Gillum *et al.* (1984) Isolation of the *Candida albicans* gene for orotidine-5'-phosphate decarboxylase by complementation of *S. cerevisiae ura3* and *E. coli pyrF* mutations. *Molec. Gen. Genet*. 198, 179-182.
2. MacCallum *et al.* (2009) Property differences among the four major *Candida albicans* strain clades. *Eukaryot Cell* 8, 373-387.
3. Fonzi & Irwin MY (1993) Isogenic strain construction and gene mapping in Candida albicans. *Genetics* 134, 717-728.
4. Murad *et al.* (2001) *NRG1* represses yeast-hypha morphogenesis and hypha-specific gene expression in *Candida albicans*. *EMBO. J.* 20, 4742-4752.
5. Negredo *et al.* (1997) Cloning, analysis and one-step disruption of the ARG5,6 gene of Candida albicans. *Microbiology* 143, 297-302.
6. Smith *et al.* (2004) A conserved stress-activated protein kinase regulates a core stress response in the human pathogen *Candida albicans*. *Molec. Biol. Cell,* 15, 4179-4190.
7. Wilson *et al.* (1999) Rapid hypothesis testing with *Candida albicans* through gene disruption with short homology regions. *J Bacteriol* 181, 1868-1874.
8. Dennison *et al.* (2005) Gene Disruption in *Candida albicans* using a synthetic, codon-optimised Cre-*loxP* system. *Fungal Genet. Biol.* 42, 737-748.
9. Davis *et al.* (2000) *Candida albicans* RIM101 pH response pathway is required for host-pathogen interactions. *Infect. Immun.* 68, 5953-5959.
10. Noble & Johnson (2005) Strains and strategies for large-scale gene deletion studies of the diploid human fungal pathogen *Candida albicans*. *Eukaryotic Cell*. 4,298-309.
11. Maidan *et al.* (2005) Carbon source induced yeast-to-hypha transition in *Candida albicans* is dependent on the presence of amino acids and on the G-protein-coupled receptor Gpr1. *Biochem. Soc. Trans.* 33, 291-293.
12. Rocha *et al.* (2001) Signaling through adenylyl cyclase is essential for hyphal growth and virulence in the pathogenic fungus *Candida albicans*. *Molec. Biol. Cell* 12, 3631-3643.
13. Ramanan & Wang (2000) A high-affinity iron permease essential for *Candida albicans* virulence. *Science* 288, 1062-1064.
14. Cao *et al.* (2017) Global regulatory roles of the cAMP/PKA pathway revealed by phenotypic, transcriptomic and phosphoproteomic analyses in a null mutant of the PKA catalytic subunit in *Candida albicans*. *Molec Microbiol* 105, 46-64.
15. Bambach *et al.* (2009) Goa1p of *Candida albicans* localizes to the mitochondria during stress and is required for mitochondrial function and virulence. *Eukaryotic Cell* 8, 1706–1720.
16. Frohner *et al.* (2009) *Candida albicans* cell surface superoxide dismutases degrade host-derived reactive oxygen species to escape innate immune surveillance *Molec. Microbiol*. 71, 240–252.
17. Noble *et al.* (2010) Systematic screens of a *Candida albicans* homozygous deletion library decouple morphogenetic switching and pathogenicity. *Nature Genetics* 42, 590–598.
