# Supplementary material for: Non-canonical signalling mediates changes in fungal cell wall PAMPs that drive immune evasion
Source: Nat Commun. 2019 Nov 22;10:5315. doi: 10.1038/s41467-019-13298-9 (PMC6876565; doi:10.1038/s41467-019-13298-9)
Supplement: Supplementary file 3 — Description of Additional Supplementary Files [file 41467_2019_13298_MOESM3_ESM.pdf]

Description of Additional Supplementary Files

File Name: Supplementary Movie 1

Description: **First time-lapse video of BMDM interactions with iron replete *C. albicans* cells.** This movie shows the first two hours of interactions between murine BMDMs and iron replete *C. albicans* cells. It is representative of twelve movies in total (4 movies from 3 mice).

File Name: Supplementary Movie 2

Description: **Second time-lapse video of BMDM interactions with iron replete *C. albicans* cells.** This movie shows the first two hours of interactions between murine BMDMs and iron replete *C. albicans* cells. It is representative of twelve movies in total (4 movies from 3 mice).

File Name: Supplementary Movie 3

Description: **First time-lapse video of BMDM interactions with iron limited *C. albicans* cells.** This movie shows the first two hours of interactions between murine BMDMs and iron replete *C. albicans* cells. It is representative of twelve movies in total (4 movies from 3 mice).

File Name: Supplementary Movie 4

Description: **Second time-lapse video of BMDM interactions with iron limited *C. albicans* cells.** This movie shows the first two hours of interactions between murine BMDMs and iron replete *C. albicans* cells. It is representative of twelve movies in total (4 movies from 3 mice).
