# Supplementary material for: Non-canonical signalling mediates changes in fungal cell wall PAMPs that drive immune evasion
Source: Nat Commun. 2019 Nov 22;10:5315. doi: 10.1038/s41467-019-13298-9 (PMC6876565; doi:10.1038/s41467-019-13298-9)
Supplement: Supplementary file 8 — Reporting Summary [file 41467_2019_13298_MOESM8_ESM.pdf]

nature research

Corresponding author(s): Alistair J P Brown

Last updated by author(s): 2019-Oct-21

Reporting Summary

Nature Research wishes to improve the reproducibility of the work that we publish. This form provides structure for consistency and transparency in reporting. For further information on Nature Research policies, see Authors & Referees and the Editorial Policy Checklist.

Statistics

For all statistical analyses, confirm that the following items are present in the figure legend, table legend, main text, or Methods section.

n/a	Confirmed	
☐	☒	The exact sample size (*n*) for each experimental group/condition, given as a discrete number and unit of measurement
☐	☒	A statement on whether measurements were taken from distinct samples or whether the same sample was measured repeatedly
☐	☒	The statistical test(s) used AND whether they are one- or two-sided<br>*Only common tests should be described solely by name; describe more complex techniques in the Methods section.*
☒	☐	A description of all covariates tested
☒	☐	A description of any assumptions or corrections, such as tests of normality and adjustment for multiple comparisons
☐	☒	A full description of the statistical parameters including central tendency (e.g. means) or other basic estimates (e.g. regression coefficient) AND variation (e.g. standard deviation) or associated estimates of uncertainty (e.g. confidence intervals)
☒	☐	For null hypothesis testing, the test statistic (e.g. F, t, r) with confidence intervals, effect sizes, degrees of freedom and P value noted<br>*Give P values as exact values whenever suitable.*
☒	☐	For Bayesian analysis, information on the choice of priors and Markov chain Monte Carlo settings
☒	☐	For hierarchical and complex designs, identification of the appropriate level for tests and full reporting of outcomes
☒	☐	Estimates of effect sizes (e.g. Cohen's d, Pearson's r), indicating how they were calculated

Our web collection on statistics for biologists contains articles on many of the points above.

Software and code

Policy information about availability of computer code

Data collection	Cytometry, FlowJo v. 10; Microscopy, Image-J v.1.47, Openlab v 4.04; Live imaging, Volocity v.6.3.1
Data analysis	Cytometry, FlowJo v. 10; Microscopy, Image-J v.1.47, Openlab v 4.04; Live imaging, Volocity v.6.3.1

For manuscripts utilizing custom algorithms or software that are central to the research but not yet described in published literature, software must be made available to editors/reviewers. We strongly encourage code deposition in a community repository (e.g. GitHub). See the Nature Research guidelines for submitting code & software for further information.

Data

Policy information about availability of data

All manuscripts must include a data availability statement. This statement should provide the following information, where applicable:

- Accession codes, unique identifiers, or web links for publicly available datasets
- A list of figures that have associated raw data
- A description of any restrictions on data availability

The authors declare that the data supporting the findings of this study are available within the paper (and its supplementary information files).

Field-specific reporting

Please select the one below that is the best fit for your research. If you are not sure, read the appropriate sections before making your selection.

☒ Life sciences ☐ Behavioural & social sciences ☐ Ecological, evolutionary & environmental sciences

For a reference copy of the document with all sections, see nature.com/documents/nr-reporting-summary-flat.pdf

Life sciences study design

All studies must disclose on these points even when the disclosure is negative.

Sample size	n = 3 for in vitro experiments; n = 6 individuals for cytokine assays; n = 30 cells or microscopy measurements. These samples sizes were chosen in attempt to confirm statistical significance for changes of about two-fold.
Data exclusions	No data were excluded
Replication	Our attempts at replication were successful.
Randomization	No randomization. Most experiments involved microbial populations that were then split into experimental and control samples.
Blinding	Experiments were not blinded. Some observations were replicated independently by different individuals.

Reporting for specific materials, systems and methods

We require information from authors about some types of materials, experimental systems and methods used in many studies. Here, indicate whether each material, system or method listed is relevant to your study. If you are not sure if a list item applies to your research, read the appropriate section before selecting a response.

Materials & experimental systems

n/a	Involved in the study	
☐	☒	Antibodies
☒	☐	Eukaryotic cell lines
☒	☐	Palaeontology
☐	☒	Animals and other organisms
☐	☒	Human research participants
☒	☐	Clinical data

Methods

n/a	Involved in the study	
☒	☐	ChIP-seq
☐	☒	Flow cytometry
☒	☐	MRI-based neuroimaging

Antibodies

Antibodies used	Fc-Dectin-1 provided by Gordon Brown
Validation	n/a

Animals and other organisms

Policy information about studies involving animals; ARRIVE guidelines recommended for reporting animal research

Laboratory animals	mice, C57BL/6, male, seven weeks old
Wild animals	n/a
Field-collected samples	n/a
Ethics oversight	University of Aberdeen Animal Welfare and Ethical Review Body

Note that full information on the approval of the study protocol must also be provided in the manuscript.

Human research participants

Policy information about studies involving human research participants

Population characteristics	Healthy volunteers provided blood samples
Recruitment	Sought by email locally
Ethics oversight	College Ethics Review Board of the University of Aberdeen

Note that full information on the approval of the study protocol must also be provided in the manuscript.

Flow Cytometry

Plots

Confirm that:

☒ The axis labels state the marker and fluorochrome used (e.g. CD4-FITC).

☒ The axis scales are clearly visible. Include numbers along axes only for bottom left plot of group (a 'group' is an analysis of identical markers).

☒ All plots are contour plots with outliers or pseudocolor plots.

☒ A numerical value for number of cells or percentage (with statistics) is provided.

Methodology

Sample preparation	Candida albicans cultures prepared as described
Instrument	BD Fortessa flow cytometer
Software	FlowJo v. 10
Cell population abundance	n/a
Gating strategy	Microbial populations were simply gated on the basis of the scatter plot (FSC/SSC), and the gated population analyzed for AF488 or CFW fluorescence intensity. The axes are clearly visible in the Supplemental Figure, and these axes remained unchanged throughout.

☒ Tick this box to confirm that a figure exemplifying the gating strategy is provided in the Supplementary Information.
